# A Review of Digital Cognitive Behavioral Therapy for Insomnia (CBT-I Apps): Are They Designed for Engagement?

**DOI:** 10.3390/ijerph18062929

**Published:** 2021-03-12

**Authors:** Begum Erten Uyumaz, Loe Feijs, Jun Hu

**Affiliations:** Faculty of Industrial Design, Eindhoven University of Technology, 5612AE Eindhoven, The Netherlands; l.m.g.feijs@tue.nl (L.F.); j.hu@tue.nl (J.H.)

**Keywords:** insomnia, cognitive behavioral therapy, online, engagement, design elements

## Abstract

There are different ways to deliver Cognitive Behavioral Therapy for Insomnia (CBT-I), of which in-person (face to face) is the traditional delivery method. However, the scalability of in-person therapy is low. Digital Cognitive Behavioral Therapy for Insomnia (dCBT-I) is an alternative and there are tools on the market that are validated in clinical studies. In this paper, we provide a review of the existing evidence-based CBT-I apps and a summary of the published usability-oriented studies of these apps. The goal is to explore the range of interaction methods commonly applied in dCBT-I platforms, the potential impact for the users, and the design elements applied to achieve engagement. Six commercially available CBT-I apps tested by scientifically valid methods were accessed and reviewed. Commonalities were identified and categorized into interactive elements, CBT-I-related components, managerial features, and supportive motivational features. The dCBT-I apps were effectively assisting the users, and the type of interactions promoted engagement. The apps’ features were based on design principles from interactive product design, experience design, online social media, and serious gaming. This study contributes to the field by providing a critical summary of the existing dCBT-I apps that could guide future developers in the field to achieve a high engagement.

## 1. Introduction

Mental and physical fitness plays an essential role in individuals’ quality of life. Approximately 25% to 30% of adults experience problems with sleep at some phase in their life caused by lifestyle-related issues [1]. People with chronic insomnia experience problems with initiating, maintaining, or obtaining restorative sleep, at least three times a week for more than three months [2]. Common issues encountered after a sleepless night are reported to be irritability, memory problems, and concentration problems [3]. 

In general, lifestyle-related and psychological factors trigger insomnia [4]. Diet, caffeine and alcohol consumption, irregular sleep hours, and an inappropriate bedroom environment may influence sleep duration and quality. Moreover, sleep anxiety or worrying about not falling asleep might have a strong contribution to restless nights even when adopting proper lifestyle habits. Dysfunctional thoughts cause a self-triggering process, and the mind gets into an everlasting state and disrupts sleep. In the literature, this state is described as the “vicious cycle” [1].

Insomnia is a treatable disorder [5]. Cognitive behavioral therapy for insomnia (CBT-I) is considered the gold standard behavioral treatment by American and European guidelines [6]. As described by its name, CBT-I aims to adjust daily habits, train the body to relax, and establish realistic and healthy associations to optimize sleep quality [7]. 

**Figure 1:**
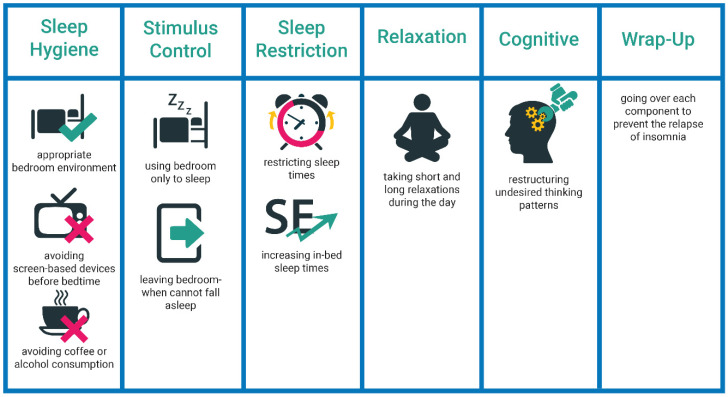
The structure of CBT-I components in in-person treatment.

CBT-I is structured as a multi-component intervention [7] with sleep hygiene education, stimulus-control, sleep restriction, relaxation training, and cognitive therapy components. See Figure 1 for an overview.

Sleep Hygiene Education: Patients are educated on sleep-related habits [8]. Through this education, the patients are informed about the appropriate conditions of a bedroom environment for a good sleep, daily habits (e.g., not watching TV before bedtime), and dietary facts (e.g., limited coffee consumption).

Stimulus-Control: Patients are informed about the importance of establishing a healthy and strong relationship between sleep and the bedroom environment. For instance, if sleep does not occur within 15 min, patients should leave their bedroom environment, engage in relaxing activities (i.e., reading a book or breathing exercise) and return when they become exhausted. 

Relaxation: Patients are introduced to a set of relaxation exercises that help overcome physical and cognitive arousal and are advised to apply them daily [3]. The most common relaxation exercises are progressive muscle relaxation, deep breathing, and imagery training exercises [2].

Sleep Restriction: Patients are asked to restrict their sleep time according to their average sleep duration of the previous week(s). Prescription quality is dependent on regular and accurate diary documentation [3]. The feasibility of the schedule is considered when prescribing a restricted-sleep schedule. The duration is extended in the upcoming weeks depending on patients’ progress and sleep efficiency or SE (i.e., a measurement of sleep quality, total sleep time divided by total time in bed). The ultimate goal is to reduce arousal and increase sleep pressure [2].

Cognitive Therapy: Cognitive therapy is focused on recognizing and altering dysfunctional thoughts and negative thinking patterns about sleep, for instance, worrying [3]. Then a set of methods are applied to overcome undesired sleep-related thoughts and unrealistic worries. The three common methods are cognitive restructuring, paradoxical intention, and thought stopping [3]. 

Evaluation and Relapse Prevention: In the last session, a recap is provided on all the components, and post measurements are carried out to evaluate the outcome. The goal is to prevent insomnia-related complaints from relapse.

### 1.1. Digital Cognitive Behavioral Therapy for Insomnia

Traditionally, a therapist guides the traditional way to deliver insomnia over weekly sessions and post-session assignments [4]. There are also other ways to deliver CBT-I, such as telephone conversations [9], workshops [10], self-help materials [11], and digital platforms [12]. 

Digital CBT-I (dCBT-I) is considered the ideal alternative to the traditional method. It is available as an online platform where the content is provided to rich media interactions and tailored (algorithm-based) information [12,13]. There are different versions of digital CBT-I: Digital CBT-I as support (provided as an auxiliary tool for in-person treatment to carry out post-session assignments), guided digital CBT-I (digital medium is provided with a therapist’s decision on the content and feedback), and fully automated digital CBT-I (digital programs that use rich media interactions and are tailored by algorithms) [8]. 

**Figure 2:**
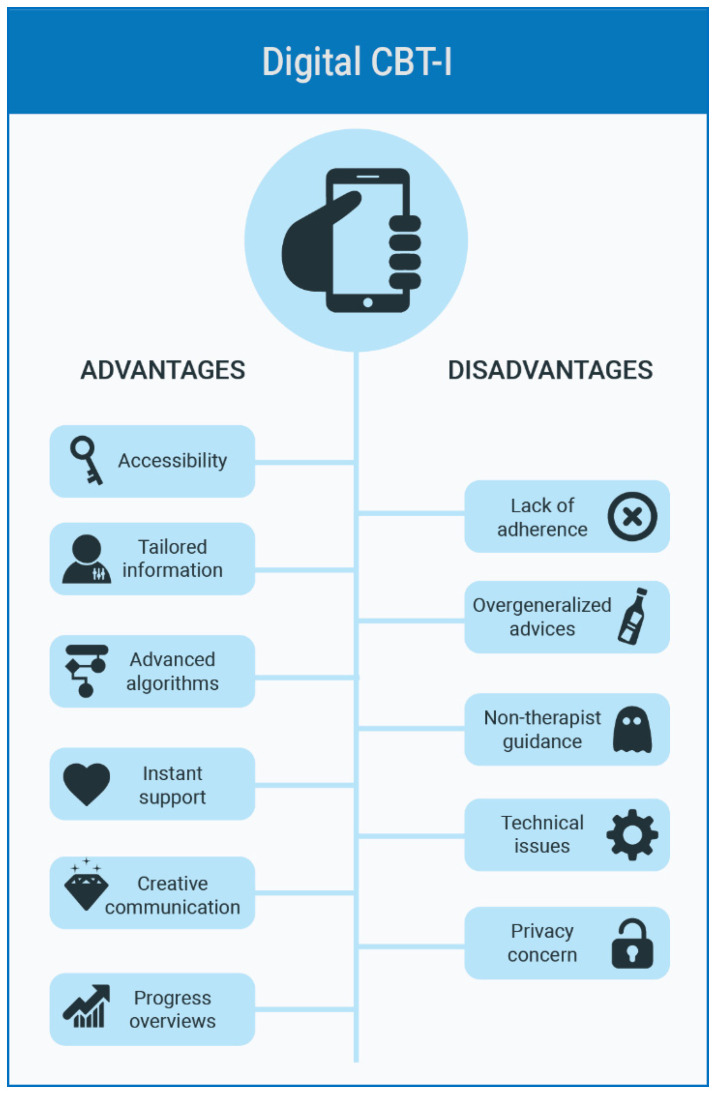
Advantages and disadvantages of digital cognitive behavioral therapy for insomnia (dCBT-I).

Figure 2 illustrates the advantages and disadvantages of dCBT-I platforms. The benefits of digital CBT-I are instant support, flexibility with time, information tailoring, and animated interactions. Furthermore, visual elements, creative interactions, record summaries, and progress evaluation reports make it appealing for the users [8,12,14]. However, digital CBT-I requires a great deal of willpower and self-discipline. Therefore, lack of adherence and non-therapist guidance are major drawbacks, next to other issues such as overgeneralized advice, technical issues, and privacy concern [12,15].

There are many dCBT-I systems on the market, yet only a scanting number of these platforms have been validated with formal or clinical validation studies [12]. According to a meta-analysis study by Seyffert et al., the insomnia severity index (ISI) [16] is decreased by 4.3 points (*p* = 0.017), sleep-efficiency (SE) (total sleep time divided by time in bed) is increased by 7.2% (*p* < 0.001), and total sleep time (TST) is increased by 20 min (*p* = 0.004), wake after sleep onset (WASO) decreased by 20 min, sleep onset latency (SOL) is decreased by 11 min [17]. According to the meta study by Trauer et al. it was found that there is a 9.91% improvement on SE, 7.61 min increase on TST, 19.03 decrease on SOL, and 26 mins decrease on WASO [18]. 

The majority of the scientific evidence and emphasis is given on clinical effectiveness; however, the problem of adherence continues to exist for the present dCBT-I platforms. Additionally, many of the scientific studies are efficacy-oriented, carried out with highly educated people who are self-motivated and have a high affinity with technology [19]. Therefore, perseverance plays a vital role in a desired treatment outcome of dCBT-I support design and interaction style facilitate the desire to engage with the platform [14]. 

Understanding the notion of *engagement* is important; it is the degree of involvement or the quality of user experience with people, services, or tangible objects [20]. Engagement is related to user experience that is appraisal of feelings triggered by the style of communication, visuals, time and behavior [14]. Engagement is essential for the success of all kinds of digital behavioral change interventions including dCBT-I [20]. After describing the methodology and results of the review study, it shall be connected to the design elements or strategies that are embedded in the platforms which in turn link to engagement and overall experience.

### 1.2. Study Objectives

This study aims to explore the content and interaction styles of commercially available dCBT-I platforms. To fulfill the goal, we have:

- Reviewed the existing full-component dCBT-I platforms and produced a detailed content summary,
- Reviewed and summarized published user studies carried out with dCBT-I platforms.

The ultimate goal is to understand: (1) which features are commonly applied on CBT-I platforms? (2) what do user studies indicate about the potential impact and value of the users’ platforms? (3) which kind of elements are commonly applied for adherence?

## 2. Materials and Methods 

We carried out a search in Google Scholar with the keywords (cognitive AND behavioral AND therapy AND insomnia AND (online OR electronic OR digital OR mobile).

A set of studies came upon targeting effect (and efficiency) and user values (feasibility, usability and perceived impact). Additionally, we have checked the references of an up-to-date review and meta-review studies related to the topic [8,17,18,21]. Then, we searched commercially available platforms on Google Play (Google LLC, Mountain View, CA, USA) and App Store (Apple Inc., Cupertino, CA, USA).

The inclusion criteria were (1) having adults (people who are aged above 18) as target users, (2) offering a full CBT-I program with components described in the introduction section of this article, (3) the effect, efficiency or feasibility studies are published in peer-review journals and sleep congresses, and (4) active in recent times (2016–2019) and available to be accessed on a digital market. 

Six different CBT-I platforms were commercially available with at least a scientifically valid study appeared from the research. Then we searched for the apps on Google Scholar to access further studies. 

**Figure 3:**
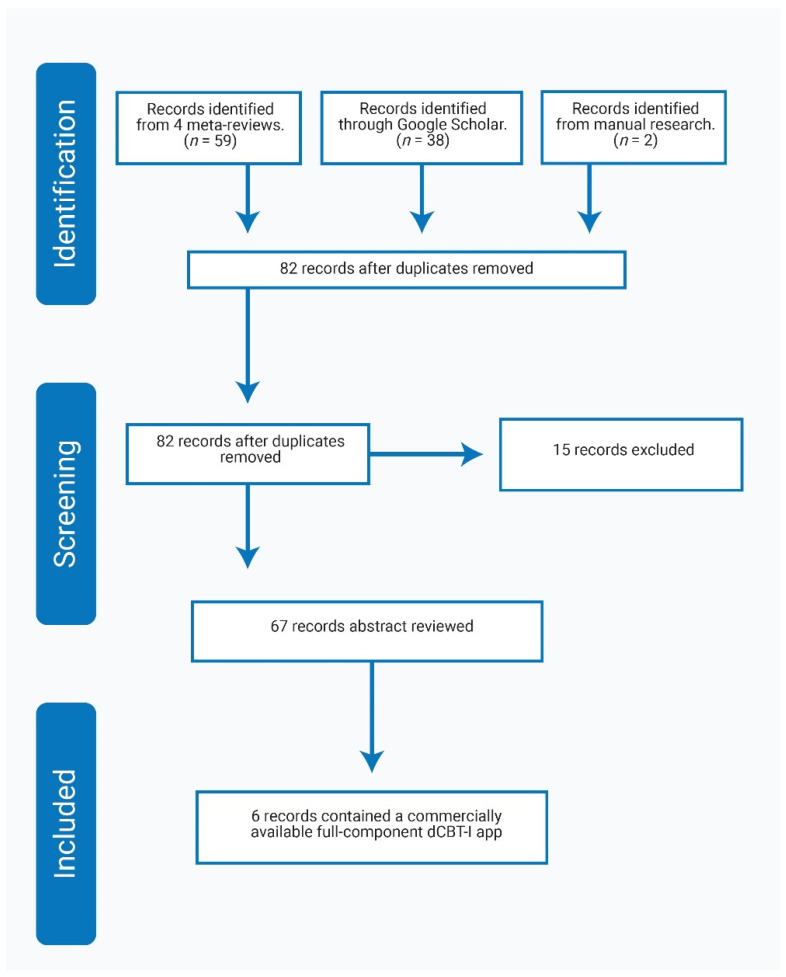
Prisma FLOW diagram for the systematic review.

Overall, there were 12 published studies: Six of them were feasibility studies [22,23,24,25,26,27], and six were clinical effect studies [14,28,29,30,31,32]. Figure 3 gives an overview of the systematic search process.

App Review: The researcher installed each platform on an Android or Apple device started using it on a daily basis, filled the sleep diaries, took the intake assessments, and reviewed each component. Notes and screenshots were taken during this evaluation, and the information is organized as *presentation style*, *available queries*, *type of feedback*, *graphical summary*, *content delivery*, *guidance*, and *support*. The list is developed based on the summaries provided in [12,14]. Additionally, a review report is written based on the insights that the researcher has gained for each platform it terms of its advantages and disadvantages. The notes were then reviewed by a technology expert who is specialized in design for coaching-based health products.

User Study Report Review: A second review study is carried out based on feasibility and user aspects of each platform with the information gained from the published studies studies [22,23,24,25,26,27]. Each article is read by the researcher, notes were taken and then summaries were produced.

## 3. Results

### 3.1. App Review

Table 1 lists the existing commercially available dCBT-I apps, the type of support, the provided information and the existing studies. Advantages and Disadvantages are listed in Table 2.

(1) Sleepio: The platform is available in English, and it is a web-based application (mobile version is also available), delivering fully-automated CBT-I support.

Presentation Style: The information is divided into modules; each module is made accessible every other week. Each module starts and ends with an overview of the topics. The information is provided with interactive quizzes and animation videos (flat design visual elements). The story is explained by a character (The Prof). In the first module, the Prof gets input from the user about what he/she wants from the therapy. Additionally, the user can follow the platform’s website’s goals and progress.

Tracking and Feedback: The sleep diary is in a survey form, including several questionnaires: Sleep quality (in-take), goal-setting (beginning), activity planner, and thought checker. Graphics (bar charts) summarize periodic measurements on sleep diary, sleep efficiency, time asleep, time in bed, and sleep quality.

CBT-I Components (CBT-I Elements): These components are sleep hygiene, stimulus control, sleep restriction, relaxation, cognitive therapy, and relapse prevention. The content is presented in animation videos and there are topics such as sleep facts, commitment, sleep efficiency, lifestyle and diet, food, bedroom factors, a simple pop-quiz to engage the users, intrusive thoughts, and the rationale behind the thought challenge strategy is explained. Then, the underlying principles of sleep restriction are explained in another module. Stimulus control and sleep restriction are described. Cognitive therapy is explained over two different modules: One for challenging thoughts and the other for racing thoughts. Negative thoughts are described, and techniques for overcoming negative thoughts and worries (diary) are explained. The app provides feedback on the types of intrusive thoughts users have about sleep (planning, sleep, awareness). There is an interactive quiz about daytime functioning, perceived sleep, and what is learned in the module. The users graduate in the last module and receive a digital completion certificate by email. 

Guidance and support: Sleep efficiency is calculated for each diary entry. Podcasts are available on progressive muscle relaxation and autogenic training. There is also a pre-bed routine activity planner. Personalized feedback is provided on the type of intrusive thoughts about sleep. There are notifications for bedtime and an online forum with the community.

Advantages and Disadvantages: The content is divided as modules and consistent with the traditional method. The information is explained with animated videos and a visually pleasing user interface. There are summaries and recaps in the beginning and end of every module. The user is rewarded with a virtual certificate when completing all the modules.

The cognitive components (i.e., benefit of journaling) are explained with animated videos, however it could be more understandable for the user if there were more interactive elements. The explanation style is clear and concise; however, the CBT-I-related topics may lack the sufficient depth. The presentation of the content stays at the superficial level and the users might not be able to comprehend all the concepts.

(2) Shuti Platform: The platform is available in English, developed as a web platform to deliver fully automated CBT-I support.

Presentation Style: The content is divided into weekly modules (cores). The information is structured as text, graphics, and videos with interactive panels and quizzes. There are clickable elements, and extra information is delivered in pop-up panels. There are also five different personas, and their stories are explained with videos or vignette testimonials. Additionally, videos are presented with experts explaining about insomnia and CBT-I. The content is summarized at the end and is reviewed at the beginning of the next module. Homework is provided as to do tasks and these tasks are for instance filling out a sleep diary and applying daily schedules. Additionally, there is an online forum where people can interact with the other users. 

Tracking and Feedback: A sleep diary is available as a survey form. Other surveys are available for sleep concerns, personal information, sleeping difficulties, sleepiness scale, inner-clock tendencies, sleep beliefs, tension scale, and health habits. Sleep diary information is summarized as bar chars that include time in bed, time asleep, sleep window, and sleep efficiency. Additionally, there is a chart on bedtime, arise time, and sleep quality. There is also a calendar view where the days the sleep diary is filled are marked.

CBT-I Components (CBT-I Elements): These components are sleep hygiene training, stimulus control, sleep restriction, relaxation training, cognitive therapy, and relapse prevention:

*(a)*  Sleep Hygiene: CBT-I treatment is introduced through a video presented by an expert, explaining the benefits of the therapy and the prevalence of insomnia. The population at risk is explained, including their gender, emotions, thinking style, medical problems, psychological problems. The impact of insomnia is explained through an interactive checklist about daytime energy, feelings, or mood. Furthermore, there is an interactive sleep hygiene game to spot issues in a hypothetical bedroom; one of such issues, for instance, introducing caffeinated drinks.
*(b)*  Sleep Scheduling: Each module starts with reviewing the sleep diary (total sleep time, total time in bed, and sleep efficiency), and then the user adjusts the arousal time based on preference. The notion of sleep restriction and sleep efficiency and stimulus control are explained in detail. There is also an interactive pop-up panel where the users can adjust the bedtimes and the sleep window to comprehend sleep efficiency. In another pop-up panel, common concerns about sleep restriction therapy and possible negative experiences are interpreted.
*(c)*  Cognitive Therapy: Common thinking errors are explained with interactive exercises, like other peoples’ opinions or personal records of thoughts and beliefs about sleep. Furthermore, strategies to avoid negative thoughts are explained in detail. Additionally, there is an interactive panel that describes the possible reasons for relapse and provides advice on coping with bad nights.

Guidance and Support: Sleep restriction is calculated from the sleep diary and it is adapted every week. There are email reminders to fill out the sleep diary. Pieces of advice are provided on establishing a pre-sleep routine. 

Advantages and Disadvantages: The information is organized as modules and delivered weekly with summaries and recaps. Interactive games and panels help explain abstract concepts such as sleep efficiency. The content is rich with satisfying explanations. However, each display contains too much text where the level of information provided might be overwhelming for users.

(3) Sleeprate App: The platform is available in English, developed as a mobile app to deliver fully automated CBT-I support.

Presentation Style: The information is presented through interactive videos, regular assessments, and informative visualizations. There are animative videos with a character who explains the concepts. Sleep diary and information summary are comprehensive. The design and the layout of the elements are visually pleasing. Reminders are available.

Tracking and Feedback: Sleep information is obtained through actigraphy and sound recording sensors of the smart device that collect information about sleep time, nap, caffeine, alcohol consumption, and smoking. There are graphical reports on bedtime-wakeup time, sleep stages, sleep efficiency and stress level.

CBT-I Components (CBT-I Elements): These components are sleep hygiene training, stimulus control, sleep restriction, relaxation, and relapse prevention. Sleep related concepts such as circadian rhythm, sleep drive, sleep regulators, the tension on sleep, and perception of sleep are explained. The influence of alcohol and caffeine use on sleep is explained. By the end of the program, the sleep diary includes several sleep hygiene-related questions. 

Guidance and Support: The platform apply the standard rules of sleep restriction and provides weekly guidance. In the first week, seven days of information recording are required for the app to provide an assessment. Wake up time is calculated the information in sleep diary. Explanatory videos (breathing techniques) are available. There are also progress summaries (including SE calculation) and reminders.

Advantages and Disadvantages: Collects information from multiple sensors (sleep diary, voice recorder) before offering an assessment program. However, the app does not provide the opportunity to fill the absent days in the first week before making an assessment program and it might mislead users. Additionally, the app sends too many reminders, that might be redundant and annoy the users. Additionally, the app is too much focused on the behavioral components and a little emphasis is given on cognitive aspect of the insomnia.

(4) CBT-I Coach: The platform is available in English, developed as a mobile app to support in-between sessions of traditional CBT-I delivery.

Presentation Style: The information is presented as text and graphics, organized in four tabs: My Sleep, Tools, Learn and Reminders. There is a checklist in the tools tab that includes a possible range of activities to organize sleep and to form daytime habits. Reminders can be set for daytime and bedtime activities.

Tracking and Feedback: A survey-based sleep diary is present. Progress summaries are available. Additionally, an intake questionnaire is available to assess the severity of insomnia. 

CBT-I Components (CBT-I Elements): These components are sleep hygiene training, stimulus control, sleep restriction, relaxation, cognitive therapy, and relapse prevention. The “Learn” tab is devoted to a basic level of sleep medicine information, sleep habits, sleep efficiency and stimulus control. A goal-setting based guidance is available for caffeine use. An audio is available to guide wind-down and a checklist is available on possible ways to change perspectives. Activities on breathing, winding down, observing thoughts, and guided imagery are explained with videos. There are also examples for changing perspectives, worrying about sleep, thinking about trauma. A yes/no checklist is available to wrap up the possibilities on a set of issues that one might have trouble with. These issues are for example having difficulty waking up in bed, worrying, and limiting caffeine use. 

Guidance and Support: A checklist is available on bedtime activities. There are video-guided breathing exercises. Sleep efficiency and recommended time for sleep are calculated. Reminders are available.

Advantages and Disadvantages: There is a rich amount of advices on possible activities during bedtime and wake time. The information is tailored based on a checklist. There is also a rich content to support relaxation and guided training. Sleep efficiency and stimulus control are explained; but sleep restriction guidance does not exist (probably because the app is an in-between support). For the Android version, some bugs prevent logging the sleep diary. The user interface is old-fashioned compared to other CBT-I apps.

(5) Night Owl: The platform is available in English, developed as a mobile app to deliver fully automated CBT-I support.

Presentation Style: Information is organized in tabs such as Today’s tasks, Completed content, Education preview, Sleep charts, Edit logs, and About. Today’s tasks is an interactive checklist about what needs to be done. In the Completed content tab there is a list of completed trainings planned to be finished in 56 days. Additionally, a list of articles and videos are available.

Tracking and Feedback: A survey-based sleep diary is available. There are additional ratings on daily stress and mood. The Edit logs tab provides a shortcut for sleep logs. Instructions are available for filling out the sleep diary (Education preview tab). Bar charts are produced for sleep efficiency, total sleep time, sleep onset latency, wake after sleep onset, number of awakenings, sleep quality rating, stress rating, and sadness rating.

CBT-I Components (CBT-I Elements): These components are sleep hygiene training, stimulus control, relaxation training, sleep restriction, and cognitive therapy. There are articles and videos explaining basic sleep training such as circadian rhythm. There are videos and articles about cognitive therapy, vicious cycle, and sleep conditioning.

Guidance and support: There are guidance on daily tasks. Sleep efficiency is calculated. Information about sleep restriction and stimulus control is provided. Additionally, a recommended bedtime is calculated. Additionally, there are progress and measurement summary on SE values. 

Advantages and Disadvantages: There is a rich and informative content especially on sleep education training. However, the content is more focused on delivering sleep biology information than supporting sleep management. The app needs a more user-centered communication perspective, and the explanation might be too technical for the users. Additionally, there are technical issues on the recent Android version of the software in accessing sleep logs. The user interface can be improved, to provide a better experience to the users.

(6) Minddistrict: The platform is available in Dutch and Georgian which is also available as a mobile app to deliver fully automated CBT-I support. The platform is also available for clinical monitoring, and user input can be viewed by a personal therapist and the family doctor.

Presentation Style: Information is presented as text, images, and graphics. Topics are organized as six modules that are delivered in the consequence of introduction, necessary information, fears, sleep habits, relaxation, and thought challenge. Stories of two different profiles (personas) are presented. There are interactive panels that prompt queries to users and expect to be answered (e.g., How do you feel about your sleep?). Additionally, goal-setting, planning, and advice features are available. 

Tracking and Feedback: The app contains two different sets of diaries on sleep and thoughts. The sleep diary records sleep and wake times during the night, subjective energy level, coffee and alcohol consumption, and other possible influences. The thought record diary helps users to challenge their thoughts. It contains questions of what happened, images, thoughts, feelings, not helpful thoughts, advantages, disadvantages, and the effect of helpful thought. Email notifications are sent to reminding assignments.

CBT-I Components (CBT-I Elements): These components are sleep hygiene training, stimulus control, sleep restriction, cognitive therapy, and relapse prevention. There are explanations about sleep stages as well and advice on sleep, daytime habits and diet consumption. Furthermore, there is guidance on planning the bedtimes. There is a module about sleep-related thoughts and vicious cycle models in which the information is explained with examples. By the end, a recap is provided about what is learned from online training to help preventing a potential relapse.

Guidance and support: Email reminders are present. Interactive dialogues prompt users to provide feedback. Additionally, a therapist can intervene if necessary.

Advantages and Disadvantages: There are interactive panels with questions in each module that encourage users to participate and set goals. Relaxation exercises are informative and guiding. Additionally, login is provided for thought challenging and for sleep information. However, the platform is limited in providing feedback and graphical summaries.

### 3.2. User Studies

We found five studies addressing three apps. A brief summary is in Table 3. We give more details of these studies below:

CBT-I Coach: CBT-I coach was developed by VA/Stanford/DoD targeting veterans (VA = U.S. Department of Veterans Affairs, DoD = Department of Defense). A survey investigation was carried out regarding the perception of the CBT-I Coach app with 138 VA trained CBT-I clinicians who has experience with treating 3.36 insomnia patients per week [22]. According to the survey, the clinicians claimed that CBT-I Coach increased patient knowledge of sleep-related facts, therapy outcome, homework compliance, and adherence. In another study involving 108 VA trained CBT-I clinicians who has experience with treating 5 insomnia patients per week [23], the perception of CBT-I Coach was further explained. It was found that 83% of 108 participants were positive about using the app in the clinic and claimed that the app increased user engagement and homework compliance.

Shuti: The Shuti platform was tested [33] with 39 participants (33 met DSM-5 criteria for major depressive disorder, and 13 reported to have cognitive complaints) over six weekly sessions and then a six-month follow-up. The investigators derived a set of benefits, challenges and perceived effects. *Benefits:* For more than half of the participants, Shuti was a solution to their problems, prepared them to cope with their future. Moreover, the app would help participants understand the factors that can influence sleep, develop good habits and challenge cognitive misperceptions. *Challenges:* The investigators showed that there were difficulties in engaging with sleep restriction advice. Especially it was a challenge to adhere to the sleep window. Moreover, some participants found issues with homework overload, restriction of sleep and overloaded text. *Side Effect:* The participants felt insecure about filling out the sleep diary manually based on estimation (avoiding checking clock). Furthermore, not being able to complete modules or diaries was associated with a sense of failure. 

In [19], 10 participants completed 6 modules of a dCBT-I program with email assistance by the therapists and filled out a satisfaction survey in the end. The participants scored higher on ISI than 7 and were scored below 14 on DASS-21 depression scale. According to the participants, the content was presented with interactive activities. The participants particularly found the sleep diary to be informative for understanding their situation. The least liked feature is the sleep restriction component. The participants advised to reduce the content and increase opportunities to contact with therapists.

Sleepio App: A trial study was conducted with 164 participants who are UK citizens above 18 years and met the criteria for persistent insomnia disorder. According to the results, it was found that attrition (dropout) rates were low (12% to 20%) compared to other studies (33% to 49%). The authors suggested that interaction components as graphics, animations, reminders, and tailored recommendations reduced dropout rates [26].

## 4. Discussion

The goal of this paper is to review the content and the interaction styles of commercially available dCBT-I platforms to tackle several research questions. 

*RQ (1): Which kinds of features are commonly applied on CBT-I platforms?*

In the existing dCBT-I apps, different sets of features are applied. These features could be categorized as: (1) interactive elements such as text, graphics, video and interactive panels; (2) CBT-I related components such as educative materials (articles and videos), sleep diary, intake surveys, podcasts and audio exercises, SE calculations, and sleep time recommendation; (3) managerial features such as goal-setting, to-do, checklists, homework and assignments, (4) supportive—motivative features such as tailored the content, online community, message and email reminders, completion certificate, progress measurements, avatars, and personas. 

*RQ (2): What do the user studies indicate about the potential impact and value of the app for the users?*

The user studies indicate that CBT-I apps are a good assistance for the users at home. Furthermore, the favored feature is found to be the sleep diary. Additionally, the interactive components such as graphics, animations, reminders and tailored recommendations to help improve engagement and homework compliance.

*RQ (3): Which kind of design elements are commonly applied to improve adherence?*

The common elements are sleep diaries and intake surveys. These apps bring in interactivity to improve adherence. The interactive product behavior [34] has become a prominent aspect of design, using feedback and feedforward as design elements. Moreover, checklists and reminders would help accomplish tasks, which is a well-known design element in the area of experience design [35]. Finally, online and social elements might help people feel supported which is a common design element addressed in the field of serious gaming [36,37], as “relatedness”.

Limitations of the Study: In this work, we had to rely on two sources of information (the published literature and the first author’s experience of using the apps). We did not have access to the design process the apps. However, the researcher did have a first-person experience [38,39,40,41], which was helpful to recognize the design issues of the six apps reviewed. We are also a new commercially available dCBT-I platforms may have appeared afterwards this study is carried out. 

## 5. Conclusions

The first conclusion is that there is a significant market for dCBT-I apps, and there are a variety of competing apps. All the six apps were reviewed in a systematic manner. We gave an overview of the content, presentation style, advantages and disadvantages of dCBT-I. We found many commonalities which we could categorize in four levels (interactive elements, CBT-I components, managerial features, and supportive motivational features). 

The CBTI components seem to be a stable common core across apps, whereas there are much more considerable variations in the other levels, most notably in the interactive elements. On this point, it is essential to note that the amount of investment influences service quality and accessibility. Some of the apps are produced by industrial organizations (i.e., Sleepio and Shuti) and others by research institutions (i.e., CBT-I Coach). Therefore, some of the platforms are freely available, yet the others cost money. 

In our study we focused on (almost) full package dCBT-I apps, whereas according to the stepped care model (Espie et al. [12]), there is also need for brief therapy apps. Although we recognize this limitation of our study, we believe that the design-for-engagement principles are useful for brief therapy apps too. It would be an interesting study to focus on adherence and engagement of brief therapy apps. 

We recognize several contemporary design principles that clearly had been put into action by turning them into supportive and motivational features (design principles from interaction design, experience design, and serious gaming). The potential impact was mostly limited in the improvement of sleep time and sleep quality according to CBT-I empirical studies. However, approaching only with this impact will not fulfill the purpose of dCBT-I platforms. The existing apps have a whole “arsenal” of features which are designed to increase engagement and adherence. This is where the real progress is happening.

Insomnia is a condition where behavior change is needed, yet very difficult to achieve. The point is that the recommendations, exercises, and so on (aimed at behavior changes which in turn are to improve sleep) are only effective when the user really follows them. It does not help to provide the most perfect recommendation and provide it with the greatest accuracy if the user, in his/her busy daily life, does not pay enough attention to it. Therefore, we have produced a detailed summary regarding the content and interaction styles of existing commercially available dCBT-I apps to explore factors related to engagement. The design elements and interaction styles emerging from our results could be used as a guideline for researchers and designers assisting them to develop products targeting a high engagement in digital behavioral change interventions.

## Figures and Tables

**Table 1:** Commercially available dCBT-I platforms, support type, presentation style, CBT-I Elements and the existing published studies.

Published Studies	[26,27]	[25,28]	[29]	[22,23,24,33]	[30]	[31,32]
CBT-I Elements	SHT, SC, SR, RT, CT and RP.	SHT, SC, SR, RT, CT and RP.	SHT, SC, SR, RT and RP.	SHT, SC, SR, RT, CT and RP.	SHT, SC, SR, RT, and CT.	SHT, SC, SR, CT and RP.
Presentation style	Textual, graphics, animation videos, interactive quizzes, and list of summaries.	Textual information, visual and interactive pop-up panels, and vignettes.	Interactive and animative videos, visualizations, reminders.	Textual information and graphics organized in tabs.	Textual information and graphics organized in tabs, interactive checklist and videos.	The information is presented as text, picture and videos. There are interactive sections.
Support type	Fully automated	Fully automated	Fully automated	Digital CBT-I as support	Fully automated	Guided digital CBT-I
Language	English	English	English	English	English	Dutch/Georgian
Platform	Sleepio	Shuti	SleepRate	CBT-I Coach	Night Owl	Minddistrict

SHT: Sleep Hygiene Training; SC: Stimulus Control; SR: Sleep restriction; RT: Relaxation Training; CT: Cognitive Therapy; RP: Relapse Prevention.

**Table 2:** Advantages and disadvantages of the reviewed platforms.

Platform	Advantages	Disadvantages
Sleepio	The information is divided as modules, there are visually pleasing animative videos, summaries and recaps, competition certificate.	Interactivity is low, content lacking scientific depth
Shuti	Available as modules, interactive and playful games, scientifically rich explanations	Too much text
Sleeprate	Collects information from multiple sensors (i.e., sleep diary, voice recorder)	Too many reminders, discrepancy between behavioral and cognitive components
CBT-I Coach	Rich amount of advices on bedtime and waketime, information is tailored based on a checklist, a rich content is available on relaxation and content training	There is no guidance on sleep restriction, there are technical issues, old fashioned user interface
Night Owl	Rich and informative content on sleep education training	The information is too scientific and technically described, lacking user-centered communication
Minddistrict	Interactive panels are available on modules, there are relaxation exercises. Diaries for sleep and thoughts are available	Limited in feedback and graphical summaries

**Table 3:** Demographic information of the user studies.

Article/Platform	Participant Number	Mean Age (SD)	Gender	Mental Health Condition	Treating CBT-I Patients	Benefits
Kuhn et al., 2016 [22]/CBT-I Coach	138 (VA ^1^ -Trained CBT-I) clinicians	47.73 (10.86)	97 f, 41 m	N/a	3.36/week	Outcome, compliance, adherence (more)
Miller et al., 2019 [23]/CBT-I Coach	108 (VA Trained CBT-I) clinicians	48.53 years (SD = 11.56)	77 f, 31 m	N/a	5/week	Positive, engagement, compliance
Chan et al., 2017 [25]/Shuti	39 (depressed) patients	59 years (N/a)	39 m	33 major depressive disorder (DSM-5 ^2^ criteria), 13 self-reported cognitive complaints	N/a	Understand factors, challenge misperceptions (more)
Meaklim et al., 2018 [19]/Shuti	10 (sleep) patients	52.4 years (SD = 13.7)	4 m, 6 f	Insomnia Severity Scores (ISI) were (M = 17.5, SD = 4.3)	N/a	Sleep diary is informative
Espie et al., 2012 [26]/Sleepio	164 (insomnia) patients	>18 years	44 m, 120 f.	N/a	N/a	Low dropout rates

^1^ VA:. ^2^ DSM-5: Diagnostic and Statistical Manual of Mental Disorders (version five).

## Data Availability

Not applicable.
